# PReS-FINAL-2097: Evaluation of gait in children with juvenile idiopathic arthritis

**DOI:** 10.1186/1546-0096-11-S2-P109

**Published:** 2013-12-05

**Authors:** SN Baydogan, E Tarakcı, O Kasapcopur

**Affiliations:** 1 Division of Physiotherapy and Rehabilitation, Faculty of Health Science, Istanbul University, Istanbul, Turkey; 2 Division of Physiotherapy and Rehabilitation, Faculty of Health Science, Istanbul University, Istanbul; 3 Department of Pediatric Rheumatology, Medical Faculty of Cerrahpasa, Istanbul University, Istanbul, Turkey

## Introduction

Juvenile idiopathic arthritis (JIA) causes pain that may lead to posture and movement modifications and arouses muscular imbalance with reduced range of motion in the affected joints. Clinical assessment of the gait is difficult in children, because of the complexity and rapidity of movement. In children with JIA, the gait pattern is further complicated by subtle compensatory gait alterations in response to joint pain and limb deformity.

## Objectives

To examine gait in children with joint involvement in knee who suffered from juvenile idiopathic arthritis (JIA) with video-based observation gait analysis.

## Methods

30 patients (male = 9, female = 21) were included in the study. Kinematic and time distance gait parameters were measured using a 10-meter walkway with separated 4 cm stripes in white and black color and a video camera. Sagittal plane views of cases (right and left, two views) and the foreground and background views of walking (two views), a total of 4 views were recorded. After observing views in the normal speed, views were observed by using slowed down frame-by-frame viewing and the stop function. Time distance parameters and motion deviations in stance phase of gait were examined.

## Results

The mean age was 9.63 ± 2.76 years (range 2-18 years). The mean disease duration was 4.41 ± 2.16 years (range 1-9 years). Patient population consisted of 11 patients with romatoid factor (+) polyarticular arthritis, 4 patients with romatoid factor (-) polyarticular arthritis, 12 patients with oligoarticular arthritis, and 3 patients psoriatic arthritis subtype. The mean of walking speed, step length, double step length and cadence were 0.51 m/s, 0.49 ± 0.07 m, 0.99 ± 0.14 m and 101 ± 25.33 steps/min respectively. The most noticeable changes in range of motion was increase of hip internal rotation(76.7%) and knee flexion(80%). Also, genu valgus incidence was 40%, genu varum was 20% and pes valgus was 36.7%.

## Conclusion

Gait is a parameter that should be considered in children with JIA. This study reported that the time-distance variables of children with JIA were decreased according to healthy peers in the literature. In the same time, we observed some gait deviations during the stance phase in lower extremity in children with JIA. These deviations are moderate or severe increase of hip adduction, hip internal rotation and knee flexion. Also we found genu valgus and pes valgus in static posture. We have identified these problems may relate to response to pain, avoidance harm to joints and some structural changes in joints.

## Disclosure of interest

None declared.

